# Evolving attitudes toward online education in Peruvian university students: A quantitative approach

**DOI:** 10.1016/j.heliyon.2024.e30566

**Published:** 2024-04-30

**Authors:** Rubén Darío Alania-Contreras, Mely Ruiz-Aquino, Aldo Alvarez-Risco, Marisol Condori-Apaza, Aparicio Chanca-Flores, Eugenia Fabián-Arias, Mauro Rafaele-de-la-Cruz, Shyla Del-Aguila-Arcentales, Neal M. Davies, Maria Luz Ortiz de Agui, Jaime A. Yáñez

**Affiliations:** a Universidad Nacional del Centro del Perú, Huancayo, Peru; b Universidad de Huánuco. Huánuco, Peru; c Universidad Tecnológica del Perú. Lima, Peru; d Escuela de Posgrado, Universidad San Ignacio de Loyola. Lima, Peru; e Faculty of Pharmacy & Pharmaceutical Sciences, University of Alberta. Edmonton, AB T6G 2H1, Canada; f Universidad Nacional Hermilio Valdizán. Huánuco, Peru; g Facultad de Educación, Carrera de Educación y Gestión del Aprendizaje, Universidad Peruana de Ciencias Aplicadas. Lima 15023, Peru

**Keywords:** e-learning, Attitude, COVID-19, University students, Peru

## Abstract

**Introduction:**

The COVID-19 pandemic has accelerated universities' adaptation process toward online education, and it is necessary to know the students' attitudes toward this online education.

**Objective:**

To describe the evolution of the attitude toward online education among social science students at a public university in Peru in the academic year 2020, in the context of the COVID-19 pandemic.

**Methods:**

The study uses a quantitative approach, a descriptive level, a non-experimental design, and a longitudinal trend. The sample consisted of 1063 students at the beginning of the class period, 908 during the classes, and 1026 at the end of the class period. The questionnaire for data collection was the Attitude scale toward online education for university students during the COVID-19 pandemic. The data was collected using Google Forms.

**Results:**

As a result, the attitude towards online education was predominantly weak negative at the beginning (51.1 %) and during the classes (49.1 %), and weak positive (48.1 %) at the end of the class period. The changes were not significant when comparing the three moments, the levels of attitude toward, intention to adopt, ease of use, technical and pedagogical support, stressors, and need for online education (*p*-value <0.05).

**Conclusion:**

The evolution of the attitude towards online education in the sample had a non-significant positive trend. In the initial and process stages, a weak negative attitude prevailed due to the institution's inexperience and poor digital infrastructure; in the end, the attitude became weak and positive due to the adaptation and need for online education.

## 1 Introduction

In 2020, the effects of the COVID-19 pandemic generated transcendental and historical changes in the university education system worldwide [[1], [2], [3], [4], [5], [6], [7], [8], [9], [10]]. The mandatory implementation of educational technology platforms to conduct classes remotely and synchronously was necessary to cope with the suspension of face-to-face educational activities due to the risks of infection by the pandemic [[11], [12], [13], [14], [15]]. In this context, university education experienced an exponential leap in time, implementing and regulating a form of education that, in its ordinary course, would have taken decades to standardize [16,17]. Being "the positive aspect of the new panorama, the usefulness that teachers give to online tools, the negative side is manifested in the weakness for the realization of field and laboratory practices in subjects that require face-to-face attendance" [18].

Alania et al. [19] define attitude as the positive or negative predisposition and evaluative effect of an individual towards specific objective behavior; Haddock and Maio [20] agree in defining it as a psychological tendency expressed by favorable or unfavorable evaluation towards a particular entity. According to Hernandez et al. [21], attitude is composed of affective, cognitive and behavioral components, which guide a person's liking or disliking of a particular object or behavior. Attitude cannot be directly observed; therefore, its measurement results from inferences from the subject's beliefs, feelings or behavioral intentions [19]. Online education is the modality that uses internet-based technologies for learning; its advantages are flexibility, convenience and accessibility to diverse students; its disadvantages are related to the usability of technology and access to it [[21], [22], [23], [24]]. It is synchronous when teachers and students interact simultaneously through teleconferences [25,26]; it is considered asynchronous when the interaction is not immediate, and the student can decide to process the information received through specialized platforms [27,28]. Six factors influence the attitude towards online education: perceived usefulness of online education, intention to adopt online education, ease of use of online education, technical and pedagogical support of online education, stressors of online education and need for online education [19].

Regarding the attitude toward online education, Hernández et al. [21] established that students have a favorable attitude towards online learning since it favors the improvement of reasoning skills, which allows them greater effectiveness and productivity in their subjects. However, in a study by Guevara et al. [29], students considered that virtual teaching negatively affects their learning process due to the teacher's lack of personalized attention and feedback. In the same sense, Reis and Leite [30] found that "digital technologies are not used for the development of learning networks and skills, which represents an obstacle to adequate digital transformation in higher education" (p. 123). For Grande et al. [31], virtual education highlights student heterogeneity [32,33]. Espinosa and Rivera [34] mentioned that "in the context of virtual education, a space of emotional, communicative and cognitive interactions is generated" (p. 14). Estrada et al. [35] found an attitude of indifference toward virtual education in university students, experiencing dissatisfaction and limited willingness to face this type of education during a pandemic despite recognizing its usefulness, which increased the dropout rate.

Some studies affirm that the anxiety and actions of the teachers have repercussions on the student's attitudes when the association is made between the two most important internal stakeholders of the university, students and teachers. Thus, Molina and Pulido [36] state that the transformation from face-to-face to online teaching due to COVID-19 has professional and educational implications. Said et al. [37] found that students perceived teachers' anxiety for fear of contagion, impacting the teaching process. With an institutional view, Córdova et al. [38] indicate that the lack of attention and allocation of resources affected academic activities. On the other hand, Ruiz et al. [39] established that "the use of virtual environments by teachers is positively and significantly related to their attitudes towards information and communication technology (ICT) in times of the COVID-19 pandemic" (p. 316); therefore, the greater the positive attitude towards ICT, the greater the use of virtual environments.

Regarding the importance of attitude toward online education, Hernandez et al. [21] emphasize: ‘The strengthening of online education is an important challenge for institutions today. The advantages it offers students in terms of its flexibility, accessibility and ease of use are important for increasing access to education and development. For online education to become a relevant ally of educational institutions and students, it is vital to consider the attitudinal factors that favor or interfere with adopting this teaching-learning modality (p. 362).

Before the COVID-19 pandemic, the universities had limited experience and implementation of online education, mainly due to economic factors due to its public nature; the problems described deficiencies in digital infrastructure, competencies in the management of e-learning tools and methods, and an evident resistance to change in the organizational culture. These characteristics were reflected in the four faculties of the social sciences: Anthropology, Communication Sciences, Sociology and Social Work. Initially, teaching protocols and technology for online education were regulated, and e-learning platforms were experimented with. When the pandemic arrived, the transformation to digital environments was mandatory and accelerated; in the transition, the positive predisposition of teachers and students to adopt the new technologies and ensure the continuity of education was necessary. In the process, teachers and students understood that the application of the online modality would last the entire school year, and adaptation to this new normality became imperative. In this context, student desertion was significant due to disbelief towards the new modality and technological gaps that prevented adequate connectivity; on the other hand, some teachers expressed stress due to difficulties they faced in using and managing technology. It was generally perceived that this emergency measure would be of short duration.

Globally, the completion of the first academic year under the online modality was a historical event; the documentation and studies of this process are relevant legacies for posterity. This context generated the need to record and evaluate the evolution of the attitude towards online education in university students since they are the ones on whom the educational efforts of the university are focused and are the main protagonists in the learning process. Thus, the results guide the continuous improvement processes in the permanence of online mode in the university educational system after the pandemic. The study's objective was to describe the evolution of the attitude toward online education among students of the social sciences faculties of the public university chosen in the academic year 2020 in the context of the COVID-19 pandemic. It was hypothesized that the evolution of attitude towards online education had a non-significant positive trend.

## 2 Methods

### 2.1 Design

The study used a quantitative approach, descriptive level, non-experimental, longitudinal design, and trend design.

### 2.2 Population and sample

The population consisted of 1436 students from the faculties of social sciences of a public university in Peru who were enrolled in the academic year 2020 (316 in Anthropology, 374 in Communication Sciences, 363 in Sociology and 383 in Social Work). The sample size was calculated based on 1436 students, a 95 % level of confidence and a 5 % margin of error. The size obtained was 302. However, the final sample consisted of 1063 students (99 % level of confidence and 2.02 % margin of error) at the beginning of the class period, 908 students ((99 % level of confidence and 2.6 % margin of error) during the classes, and 1026 at the end of the class period (99 % level of confidence and 2.155 % margin of error). Students enrolled in the 2020 I and 2020 II periods were considered inclusion criteria; students who stopped attending or reserved their enrollment and those who did not want to participate in the study were excluded.

### 2.3 Instrument

The “Attitude to Online Education Attitude Scale for College Students in Crisis by COVID-19”, adapted from Mehra and Omidian (2012), validated and reliably tested in a previous study by Alania et al. (2022), was used as the data collection instrument. The questionnaire consists of 63 items, with five Likert-scale response options measuring six dimensions: perceived usefulness of online education, intention to adopt online education, ease of use of online education, technical and pedagogical support of online education, stressors of online education, and need for online education. For the rating of items 1, 2, 3, 4, 5, 6, 7, 8, 9, 13, 14, 15, 16, 18, 25, 26, 27, 30, 31, 32, 38, 42, 44, 45, 46, 47, 48, 50, 51, 59, 60, 61, 62 and 63 scores are assigned as follows: strongly disagree = 1, disagree = 2, undecided = 3, agree = 4, strongly agree = 5; the remaining items are reverse scored. The validity and reliability of the instrument were performed in a previous study by the authors in the same unit of analysis [19]; the content validation was performed with the judgment of 16 experts when evaluated with Aiken's V, the excellent validity of all indicators were determined, for being higher than 0.85 (*p*-value <0.008). In the same study, construct validity was performed by applying the instrument to a sample of 6852 university students; the corrected Pearson's r correlation coefficient results reported validity between sufficient (0.2–0.34) and excellent (0.55–1). In evaluating the instrument's reliability, an internal consistency coefficient of Cronbach's alpha of 0.96 was calculated, which meant excellent reliability.

### 2.4 Data collection and analysis procedure

Data was collected from May 2020 to February 2021 through online surveys by Google Forms that were applied at three points in time for the two groups of students. Group 1: The collection was at the beginning of the 2020 I classes period (May–June 2020), in the middle of the classes period (July 2020) and at the end of the 2020 I classes period (August 2020). Group 2: The collection was at the beginning of the 2020 II classes period (September–October 2020), in the middle of the classes period (November 2020), and at the end of the 2020 II classes period (February 2021). The outcomes were calculated using the sum of the participants of both groups. The responses collected with Google Forms were downloaded in an Excel file and exported to the SPSS v.26 statistical program. After exploring the responses, the dimensions' subtotals and the variable's total were computed at each of the evaluation moments, adding up the response codes of each'subject. Simple and crossed-frequency tables were elaborated to describe the results, and the chi-squared statistic was applied to compare the variable and its dimensions in the three evaluation moments. The scales were applied to obtain the levels of the dimensions and the variables. As an ethical consideration, the participants' acceptance to carry out the survey and use the data was requested using informed consent, and their anonymity was always maintained.

### 2.5 Ethical issues

Informed consent was obtained from all patients/participants for the current study. The ethical approval was N° 005-CEFMH-2022/UPLA from the Unit of Research-UPLA.

## 3 Results

### 3.1 Demographic information

Table 1 and Table 2 show the demographic information of the participants.

**Table 1:** Faculty and sex of students by observation period.

Faculty	Sex	Beginning		Process		Final	
		f	%	f	%	f	%
Anthropology	Female	87	69.0	89	66.9	84	57.1
	Male	39	31.0	44	33.1	63	42.9
Communication Sciences	Female	161	54.6	144	63.7	142	53.8
	Male	134	45.4	82	36.3	122	46.2
Social work	Female	376	95.9	307	96.5	332	96.2
	Male	16	4.1	11	3.5	13	3.8
Sociology	Female	197	78.8	185	80.1	190	70.4
	Male	53	21.2	46	19.9	80	29.6
Total	Female	821	77.2	725	79.8	748	72.9
	Male	242	22.8	183	20.2	278	27.1

Group 1: The collection was at the beginning of the 2020 I classes period (May–June 2020), in the middle of the classes period (July 2020) and at the end of the 2020 I classes period (August 2020). Group 2: The collection was at the beginning of the 2020 II classes period (September–October 2020), in the middle of the classes period (November 2020), and at the end of the 2020 II classes period (February 2021). The outcomes were calculated using the sum of the participants of both groups.

**Table 2:** Faculty and age of students by observation period.

Faculty	Age	Beginning		Process		Final	
		f	%	f	%	f	%
Anthropology	<20	40	31.7	37	27.8	40	27.2
	20 a 22	59	46.8	62	46.6	66	44.9
	>22	27	21.4	34	25.6	41	27.9
Communication science	<20	91	30.8	42	18.6	46	17.4
	20 a 22	141	47.8	127	56.2	162	61.4
	>22	63	21.4	57	25.2	56	21.2
Social work	<20	145	37.0	115	36.2	98	28.4
	20 a 22	190	48.5	166	52.2	174	50.4
	>22	57	14.5	37	11.6	73	21.2
Scociology	<20	79	31.6	49	21.2	59	21.9
	20 a 22	131	52.4	125	54.1	160	59.3
	>22	40	16.0	57	24.7	51	18.9
Total	<20	355	33.4	243	26.8	243	23.7
	20 a 22	521	49.0	480	52.9	562	54.8
	>22	187	17.6	185	20.4	221	21.5

Group 1: The collection was at the beginning of the 2020 I classes period (May–June 2020), in the middle of the classes period (July 2020) and at the end of the 2020 I classes period (August 2020). Group 2: The collection was at the beginning of the 2020 II classes period (September–October 2020), in the middle of the classes period (November 2020), and at the end of the 2020 II classes period (February 2021). The outcomes were calculated using the sum of the participants of both groups.

The students’ sex was predominantly female in the three observation periods, both in the total sample and each of the faculties, with the highest proportion in Social Work and Sociology, exceeding 57 %.

The predominant age of the students was 20–22 years in the three observation moments, both in the total sample and in each of the faculties, with a higher percentage in the faculties of Communication Sciences and Sociology. The average age of the students in the total sample was 20.77 years (SD = 2.74 years) at the beginning of the study, 21.12 years (SD = 2.62 years) during the process and 21.38 years (SD = 3.7 years) at the end of the study. This statistic showed values identical to those of the total sample in each of the faculties and each observation period.

### 3.2 Level of attitude toward online education

The variable attitude toward online education was measured at the beginning, process and end moments of the 2020 academic year (Table 3).

**Table 3:** Level of attitude toward online education.

Attitude level	Time of evaluation		
	Beginning	Process	Final
Strong negative	43 (4 %)	32 (3.5 %)	32 (3.1 %)
Weak negative	543 (51.1 %)	446 (49.1 %)	470 (45.8 %)
Weak positive	441 (41.5 %)	403 (44.4 %)	494 (48.1 %)
Strong positive	36 (3.4 %)	27 (3 %)	30 (2.9 %)
Total	1063	908	1026

Group 1: The collection was at the beginning of the 2020 I classes period (May–June 2020), in the middle of the classes period (July 2020) and at the end of the 2020 I classes period (August 2020). Group 2: The collection was at the beginning of the 2020 II classes period (September–October 2020), in the middle of the classes period (November 2020), and at the end of the 2020 II classes period (February 2021). The outcomes were calculated using the sum of the participants of both groups.

The student's attitude towards online education was predominantly weak negative at the beginning (51.1 %) and process (49.1 %), while at the end, it became weak positive (48.1 %). The strong negative attitude did not exceed 4 %, and the strong positive attitude 3.5 %. On the other hand, it is appreciated that the weak positive attitude has increased considerably, although it was not significant (*p*-value <0.05) from 41.5 % at the beginning; it went to 44.4 % in the process and reached 48.1 % at the end.

During the 2020 academic year, between the beginning and the end, the sample showed a moderate increase in the positive attitude and a relative decrease in the negative attitude towards online education; at the beginning, the weak negative attitude predominated (51.1 %), decreasing in the process (49.1 %) and at the end, the weak positive attitude predominated (48.1 %). Overall, the attitude towards online education was negative (weak + strong) at the beginning (55.1 %) and process (52.6 %), and positive (weak + strong) at the end (51 %).

### 3.3 Level of the dimensions of attitude toward online education

#### 3.3.1 Level of perception of the usefulness of online education

Table 4 shows that the perception of the usefulness of online education was also predominantly weak negative at the beginning (53.3 %) and process (47.2 %), while at the end, it became weak positive (46.6 %). On the other hand, it is observed that the weak positive perception increased very significantly (*p*-value <0.01) from 35.7 % at the beginning to 43.9 % in the process and 46.6 % at the end.

**Table 4:** Level of perception of the usefulness of online education.

Attitude level	Time of evaluation		
	Beginning	Process	Final
Strong negative	82/7.7 %)	45 (5 %)	61 (5.9 %)
Weak negative	567 (53.3 %)	429 (47.2 %)	460 (44.8 %)
Weak positive	380 (35.7 %)	399 (43.9 %)	478 (46.6 %)
Strong positive	34 (3.2 %)	35 (3.9 %)	27 (2.6 %)
Total	1063	908	1026

Group 1: The collection was at the beginning of the 2020 I classes period (May–June 2020), in the middle of the classes period (July 2020) and at the end of the 2020 I classes period (August 2020). Group 2: The collection was at the beginning of the 2020 II classes period (September–October 2020), in the middle of the classes period (November 2020), and at the end of the 2020 II classes period (February 2021). The outcomes were calculated using the sum of the participants of both groups.

There was a significant increase in the positive perception and a notable decrease in the negative perception of the usefulness of online education between the beginning and the end of the period. So, in the beginning, the weak negative perception was predominant (53.3 %), decreasing in the process (47.2 %). In the end, the weak positive perception was predominant (46.6 %).

#### 3.3.2 Level of intention to adopt online education

The intention to adopt online education was predominantly weak and negative at the beginning (44.9 %) and process (44.6 %) and weak and positive at the end (42.6 %). Firm negative intention did not exceed 7 %, and strong positive intention ranged from 9.4 % at baseline to 11.5 % at the end. There were no significant differences (*p*-value <0.05) in the intention to adopt online education between the three observation times (Table 5).

**Table 5:** Level of intention to adopt online education.

Attitude level	Time of evaluation		
	Beginning	Process	Final
Strong negative	72/6.8 %)	55 (6.1 %)	49 (4.8 %)
Weak negative	477 (44.9 %)	405 (44.6 %)	425 (41.4 %)
Weak positive	414 (38.9 %)	373 (41.1 %)	437 (42.6 %)
Strong positive	100 (9.4 %)	75 (8.3 %)	115 (11.2 %)
Total	1063	908	1026

Group 1: The collection was at the beginning of the 2020 I classes period (May–June 2020), in the middle of the classes period (July 2020) and at the end of the 2020 I classes period (August 2020). Group 2: The collection was at the beginning of the 2020 II classes period (September–October 2020), in the middle of the classes period (November 2020), and at the end of the 2020 II classes period (February 2021). The outcomes were calculated using the sum of the participants of both groups.

### 3.4 Level of ease of use of online education

The weak negative level of ease of use of online education stands out at all three evaluation moments, with 52.6 % at the beginning, 58.6 % in the process, and 48.8 % at the end. The decrease in the weak negative and the increase in the weak positive levels was highly significant (*p*-value <0.01). The negative level (weak + strong) increased from 63.4 % at baseline to 65.9 % in the process and decreased to 55.7 % at the end, while the positive level (weak + strong) decreased from 36.6 % at baseline to 34.2 % in the process and increased to 44.2 % at the end (Table 6).

**Table 6:** Level of ease of use of online education.

Attitude level	Time of evaluation		
	Beginning	Process	Final
Strong negative	115 (10.8 %)	66 (7.3 %)	71 (6.9 %)
Weak negative	559 (52.6 %)	532 (58.6 %)	501 (48.8 %)
Weak positive	339 (31.9 %)	263 (29.0 %)	390 (38.0 %)
Strong positive	50 (4.7 %)	47 (5.2 %)	64 (6.2 %)
Total	1063	908	1026

Group 1: The collection was at the beginning of the 2020 I classes period (May–June 2020), in the middle of the classes period (July 2020) and at the end of the 2020 I classes period (August 2020). Group 2: The collection was at the beginning of the 2020 II classes period (September–October 2020), in the middle of the classes period (November 2020), and at the end of the 2020 II classes period (February 2021). The outcomes were calculated using the sum of the participants of both groups.

#### 3.4.1 Level of technical and pedagogical support for online education

The weak negative level of online education's technical and pedagogical support stands out in the three moments of observation, with 53.5 % at the beginning, 58 % in the process and 52.5 % at the end. The decrease in the weak negative level and the increase in the weak positive level were also highly significant (*p*-value <0.01). The negative level (weak + strong) increased from 61.5 % at baseline to 64.1 % in the process and decreased to 56.3 % in the end, while the positive level (weak + strong) decreased from 38.8 % at baseline to 35.9 % in the process and increased to 43.6 % in the end (Table 7).

**Table 7:** Level of technical and pedagogical support for online education.

Attitude level	Time of evaluation		
	Beginning	Process	Final
Strong negative	82 (7.7 %)	55 (6.1 %)	39 (3.8 %)
Weak negative	569 (53.5 %)	527 (58.0 %)	539 (52.5 %)
Weak positive	392 (36.9 %)	304 (33.5 %)	426 (41.5 %)
Strong positive	20 (1.9 %)	22 (2.4 %)	22 (2.1 %)
Total	1063	908	1026

Group 1: The collection was at the beginning of the 2020 I classes period (May–June 2020), in the middle of the classes period (July 2020) and at the end of the 2020 I classes period (August 2020). Group 2: The collection was at the beginning of the 2020 II classes period (September–October 2020), in the middle of the classes period (November 2020), and at the end of the 2020 II classes period (February 2021). The outcomes were calculated using the sum of the participants of both groups.

#### 3.4.2 Stressors of online education

Online education stressors were predominantly weak negative levels at baseline (41 %), in-process (42.5 %), and end-line (37.3 %). There were no significant differences (*p*-value <0.05) in online education stressors between the three observation times (Table 8).

**Table 8:** Level of online education stressors.

Attitude level	Time of evaluation		
	Beginning	Process	Final
Strong negative	198 (18.6 %)	193 (21.3 %)	212 (20.7 %)
Weak negative	436 (41 %)	386 (42.5 %)	383 (37.3 %)
Weak positive	322 (30.3 %)	241 (26.5 %)	328 (32 %)
Strong positive	107(10.1 %)	88 (9.7 %)	103 (10 %)
Total	1063	908	1026

Group 1: The collection was at the beginning of the 2020 I classes period (May–June 2020), in the middle of the classes period (July 2020) and at the end of the 2020 I classes period (August 2020). Group 2: The collection was at the beginning of the 2020 II classes period (September–October 2020), in the middle of the classes period (November 2020), and at the end of the 2020 II classes period (February 2021). The outcomes were calculated using the sum of the participants of both groups.

#### 3.4.3 Need for online education

The need for online education was predominantly weak negative at the beginning (51.1 %) and in the process (49.1 %), while the weak positive level stood out at the end (48.1 %). The decrease in the weak negative level and increase in the weak positive level were highly significant (*p*-value <0.01). The negative level (weak + strong) decreased from 55.1 % at baseline to 52.6 % in the process and 48.9 % at the end, while the positive level (weak + strong) increased from 44.9 % at baseline to 47.4 % in the process and 51 % at the end (Table 9).

**Table 9:** Level of need for online education.

Attitude level	Time of evaluation		
	Beginning	Process	Final
Strong negative	43 (4 %)	32 (3.5 %)	32 (3.1 %)
Weak negative	543 (51.1 %)	446 (49.1 %)	470 (45.8 %)
Weak positive	441 (41.5 %)	403 (44.4 %)	494 (48.1 %)
Strong positive	36 (3.4 %)	27 (3 %)	30 (2.9 %)
Total	1063	908	1026

Group 1: The collection was at the beginning of the 2020 I classes period (May–June 2020), in the middle of the classes period (July 2020) and at the end of the 2020 I classes period (August 2020). Group 2: The collection was at the beginning of the 2020 II classes period (September–October 2020), in the middle of the classes period (November 2020), and at the end of the 2020 II classes period (February 2021). The outcomes were calculated using the sum of the participants of both groups.

#### 3.4.4 Comparison of attitude toward online education by faculty and attributional variables

Highly significant differences were found in the attitude toward online education in the three moments of observation in the Faculty of Anthropology (*p*-value <0.01) but not in the Faculties of Communication Sciences, Social Work and Sociology (*p*-value <0.05). Table 10 shows that in the faculties of Anthropology and Communication Sciences, a weak negative attitude predominated in the three moments of evaluation; in Anthropology, it was 56.3 % at the beginning and 48.3 % at the end, and in Communication Sciences, it was 49.8 % at the beginning and 51.5 % at the end. In the Faculty of Social Work, weak negative attitude predominated at the beginning (52.8 %) and weak positive attitude at the beginning (50 %) and end (47.2 %), while in the Faculty of Sociology, weak negative attitude predominated at the beginning (47.2 %) and process (50.6 %), and weak positive attitude at the end (53.7 %). When comparing the results by attributive variables: sex, age (under 19 years old, 19–20 years old and over 20 years old), level of studies (initial, intermediate and advanced), place of residence, family size (small, medium and large) and marital status (single and unmarried), at the three points in time, significant differences were found in the attitude of male students (*p*-value <0.05), highly significant differences in the attitude of students aged 19–20 years (*p*-value <0.01), highly significant differences in the attitude of students at the intermediate level of studies (*p*-value <0.01) and significant differences in the attitude of students at the advanced level of studies (*p*-value <0.05), significant differences in the attitude of students residing outside the city of Huancayo (*p*-value <0.05), and significant differences in the attitude of students with small families (*p*-value <0.05). In all these cases, the attitude was weak and negative in the beginning and process, and in the end, weak and positive. No significant differences were reported in the attitude of single and non-single students (*p*-value <0.05).

**Table 10:** Level of need for online education.

Faculty	Attitude level	Time of evaluation		
		Beginning	Process	Final
Anthropology	Strong negative	7.1 %	3 %	4.8 %
	Weak negative	56.3 %	56.4 %	48.3 %
	Weak positive	32.5 %	40.6 %	46.9 %
	Strong positive	4 %	0 %	0 %
Communication Sciences	Strong negative	4.1 %	4.9 %	2.3 %
	Weak negative	49.8 %	51.3 %	51.5 %
	Weak positive	42.7 %	38.9 %	43.9 %
	Strong positive	3.4 %	4.9 %	2.3 %
Anthropology	Strong negative	3.1 %	3.1 %	4.1 %
	Weak negative	52.8 %	43.4 %	44.6 %
	Weak positive	41.3 %	50 %	47.2 %
	Strong positive	2.8 %	3.5 %	4.1 %
Anthropology	Strong negative	4 %	3 %	1.9 %
	Weak negative	47.2 %	50.6 %	40.7 %
	Weak positive	44.8 %	44.2 %	53.7 %
	Strong positive	4 %	2.2 %	3.7 %

Group 1: The collection was at the beginning of the 2020 I classes period (May–June 2020), in the middle of the classes period (July 2020) and at the end of the 2020 I classes period (August 2020). Group 2: The collection was at the beginning of the 2020 II classes period (September–October 2020), in the middle of the classes period (November 2020), and at the end of the 2020 II classes period (February 2021). The outcomes were calculated using the sum of the participants of both groups.

## 4 Discussion

The results of the attitude towards online education of public university students in Peru in the context of COVID-19 show that at the beginning of the academic year 2020, the weak negative level was predominant (51.1 %). In the process, the attitude remained weak and negative. However, with a lower index (49.1 %) and at the end of the academic year, it became weak positive (48.1 %); thus, it is inferred that there was a positive tendency in the evolution of the attitude toward online education. When the hypothesis test was performed, it was determined that the changes between the beginning of the class period, during the classes and the end of the class period in the attitude toward online education were not significant; even so, it had a positive tendency. Thus, the research hypothesis was accepted.

Similar situations were found in another research. Thus, Guevara et al. [40] determined that virtual teaching has a negative impact on the learning process, according to students at the National Autonomous University of Mexico. By focusing on the participation of teachers in this process, Said et al. [37] established that students perceive teachers' problems with COVID-19 as negatively impacting learning. In the same sense, Monteiro and Leite [30] determined that students considered that the limited digital competencies of teachers hinder digital transformation. Moreover, they generate more significant digital divides; thus, Grande et al. [31] indicate that virtual education highlights student heterogeneity. The positive predisposition of students towards online education is a determining factor for successful learning, especially in a crisis such as the one generated by the pandemic. This predisposition, which can be positive or negative, manifests itself as attitude. According to Alania et al. [19] and Haddock and Maio [20], attitude is a tendency that predisposes behavior toward a goal or entity, which in the case of the present study was online education. The study also showed that it could measure attitude through cognitive, affective, and behavioral inferences [21]. It is agreed with Hernandez et al. [21] that the advantages of flexibility, convenience and accessibility of online education is related and even conditioned by the usability of the technologies used and access to them, as also stated by Monteiro and Leite [30], influencing these in the favorable or unfavorable attitude of students. It is necessary to emphasize that virtual teaching and learning were already practiced before the pandemic, with predominantly asynchronous experiences; however, in the context of COVID-19, online education, in its synchronous mode, was implemented standardized in universities worldwide.

Regarding the dimension of perceived usefulness of online education, in the initial stage, most students expressed a weak negative attitude (53.3 %) due to the lack of experience in online or virtual education in the faculties studied. Therefore, they did not have adequate infrastructure, which coincides with the study of Monteiro and Leite [30], who found that students perceive that digital technologies are not used for developing networks and learning skills. The institution tried to generate the conditions to start the 2020 academic year online through emergency measures. The process was delayed for a month and a half at the start of the 2020-I academic semester; such facts explain the weak negative attitude in students at the initial moment. However, the experiences improved in the process, gradually consolidating the technological transition of the institution and the adaptation of teachers and students. This aspect is reflected in the positive evolution trend of the dimension of perception of the usefulness of online education; in the process, the weak negative predominance was maintained, but with a lower rate (47.2 %), and at the final moment, the attitude changed to weak positive (46.6 %). In the 2020 period, the changes in the weak positive level were statistically significant and tended to increase.

About adopting online education, the weak negative attitude predominated in the beginning and process, being slightly higher (44.9 %). At that time, students considered that remote education would last only a few weeks or months, showing disbelief towards its effectiveness, which led to a high student dropout rate; many students' poor accessibility to technology and the internet deepened their negative attitude. Despite its usefulness, Estrada et al. [35] also found that the attitude of indifference towards virtual education increased the dropout rate.

To alleviate the problem, the public university provided mobile devices to a third of the student population, who accredited economic precariousness; the weak negative trend was maintained in the process (44.6 %). However, the imminence of studying the entire 2020 academic year online generated conformity that favored the acceptance of the new modality, which was reflected in the increase of the weak positive level in the final moment (42.6 %); however, the changes in the three moments were not significant. It should be noted that, due to data speed limitations, most teachers and students do not activate their cameras in the synchronous sessions, the classes being, in general, primarily auditory experiences. However, this phenomenon is not isolated. Guevara et al. [40] also found that Mexican university students consider virtual teaching negatively affecting their learning process due to the teacher's lack of personalized attention and feedback. Based on the findings, it can be affirmed that to achieve a strong positive attitude of students toward the adoption of online education, adequate conditions of connectivity, access to technology, and didactic strategies to ensure effective and quality learning must be generated; universities must improve these factors for the continuity of online education in "post-covid" times.

The ease-of-use dimension of online education refers to students' attitudes regarding the usability of the virtual tools and platforms available to the institution to develop learning. According to the results, in the initial stage, a weak negative attitude predominated in most students (58.6 %), which can be explained by the fact that most of the student and teacher population had no experience or knowledge of e-learning tools before the pandemic. Four years earlier, the public university in Peru had already included the Chamilo platform in its system; however, it was never used for learning in the social sciences faculties. To face the crisis generated by COVID-19, the University established the use of the Moodle platform for virtual classrooms and Microsoft Teams for videoconference classes; the understanding and usability of these platforms implied a learning process that for many teachers was difficult and was evidenced by the time of the process, where the weak negative attitude increased (58.6 %). At the end of the 2020-I academic semester, the university decided to discard the use of the Moodle platform, and the exclusivity of Microsoft Teams, a repository of academic material and means of evaluation, was established for class sessions. Simultaneously, the e-learning platforms were improving their usability according to the experiences and needs of global education. These factors were expressed in the significant decrease of the weak negative level at the final moment (48.8 %) and, at the same time, the significant increase of the weak positive level (38 %), although the weak negative attitude continued to prevail.

We agree with Grande et al. [31] that virtual education highlights student heterogeneity. The university should continue training teachers and students in e-learning technologies. It is also necessary to improve the usability of virtual platforms on mobile devices with designs that respond to the Latin American reality- Due to their socioeconomic characteristics and family size, which exceed the availability of computers at home, many students can only connect to learning sessions through their cell phones.

The dimension of attitude towards technical and pedagogical support of online education is related to ease of use; in the three moments of both dimensions, a weak negative attitude predominated; however, negativity is more evident in the dimension of attitude towards technical and pedagogical support in the three moments of the study: 53.5 % at the beginning, 58 % in the process and 52.5 % at the end. The characteristics of this dimension respond to structural problems that have long affected university education in Peru. Like other public universities, the public university in Peru depends economically on the State, which provides insufficient economic investment to the university sector; in this regard, Córdova et al. [38] indicated that the lack of greater attention and allocation of resources to the main local actors of the universities affects academic activities. The problem is compounded by the inefficiency of the rules governing the procurement processes of the state system, characterized by excessive red tape that slows down the management of resources for the modernization of digital infrastructure. This situation has generated a clear disadvantage for public universities in the face of the growth of private universities, which, at the time of the pandemic, had already implemented e-learning platforms and had extensive experience in their use. For the students, it was inevitable to compare the response capacity to the crisis of the technical and pedagogical supports of the public university in Peru with other universities, an assessment reflected in this dimension.

Regarding the stressors dimension of online education, the weak negative level predominated in the three moments, starting with 41 %, rising to 42.5 % in the process and decreasing to 37.3 % in the end. The negative levels of this dimension denote unfavorable predispositions to face stressful situations, being, at the same time, a limiting factor for learning achievement. The COVID-19 pandemic generated stressful situations that impacted online learning. The mandatory social confinement and the first wave of the pandemic coincided with the beginning and process measurements. The academic-related stressors were the overload of tasks, the instability of digital platforms, the limitations of speed and connectivity, the poor adaptability of some teachers to digital environments, and the weak socialization in the new environment. It became clear that the online modality has weaknesses in generating affective and emotional interaction spaces, which are transcendental competencies for learning at any stage of life.

It is necessary to collect, study, systematize and learn from positive didactic experiences that occurred in these stages of adaptation to improve the affective, emotional and motivational processes; in this sense, Espinosa and Rivera [34] determined that in online education spaces of emotional, communicative and cognitive interactions are generated. The reasons that explain these results are similar to those mentioned in adopting online education and are related to digital and economic gaps. In the dimension of the need for online education, the weak negative level predominated in the moments of beginning (51.1 %) and process (49.1 %). However, in the evaluation of the final moment, the attitude became predominantly weak positive (48.1 %); based on the results, it can be inferred that gradually, there was a positive reflection of students about the opportunities of online education, recognizing the advantages that only this modality can offer. The increasingly favorable attitude towards the continuity of online education also indicates that the university system already accepts the idea that "online education is here to stay". In that sense, Molina and Pulido [36] agree that the transformation towards online education, as a consequence of COVID-19, has considerable professional and educational implications. In 2020, science needed to study and document the transformation of the traditional educational environment to an entirely virtual one, which meant a transcendental historical process for world education. The knowledge gathered is an essential legacy for posterity; based on these studies, shortly it is possible to propose new theories, methods, and educational strategies that respond to the needs of an increasingly virtualized and technologized education, where physical spaces and distances are not obstacles for the achievement of significant learning. As protagonists and pioneers of change, the current actors of university education have an important role in the destinies of world education.

The study has certain limitations, such as the lack of control over the response environment of the participants, which could influence the quality of the data collected. Variability in the conditions under which the respondents completed questionnaires could affect the consistency of responses. Additionally, the possibility of selection bias is a concern, as participation in the survey is voluntary, which may result in a non-representative student population sample. The lack of direct contact with participants limits the ability to clarify doubts or delve into ambiguous answers. The fact that the data was collected during the pandemic period would limit its generalization in post-pandemic scenarios.

## 5 Conclusion

It was determined that the evolution of the attitude toward online education in students of the social sciences faculties of the public university in the 2020 academic year, in the context of the COVID-19 crisis, had a non-significant positive trend. It was determined that the evolution of attitude towards online education in the six dimensions: perceived usefulness of online education, intention to adopt online education, ease of use of online education, technical and pedagogical support of education online, stressors of online education and need for online education, had a positive although not significant trend.

This study was developed during a pandemic when equipment and Internet connection were limited, which could explain why the positive attitude has grown very limited. Considering that this public university has limited government resources for its infrastructure and is not located in a central city in Peru, students could, due to the resources and their families, have limited technological equipment, so the attitude towards online education is not maximized despite the known benefits. Without a doubt, in future studies in post-pandemic times where Internet connectivity and equipment have improved, it is likely that the attitude has changed and has been significantly impacted, and there may be more students who opt for this type of education in future vocational training.

## Funding

Not applicable.

## Data availability statement

The data associated with your study has not been deposited into any publicly available repository. However, the data will be made available on request.

## Ethical considerations

Informed consent was obtained from all patients/participants for the current study. The ethical approval was N° 005-CEFMH-2022/UPLA from the Unit of Research-UPLA (UPLA = Universidad Peruana Los Andes).

## CRediT authorship contribution statement

**Rubén Darío Alania-Contreras:** Writing – review & editing, Writing – original draft, Visualization, Methodology, Investigation, Formal analysis, Data curation, Conceptualization. **Mely Ruiz-Aquino:** Writing – review & editing, Writing – original draft, Visualization, Validation, Methodology, Investigation, Formal analysis, Conceptualization. **Aldo Alvarez-Risco:** Writing – review & editing, Writing – original draft, Visualization. **Marisol Condori-Apaza:** Writing – review & editing, Writing – original draft, Validation, Methodology, Investigation, Formal analysis, Data curation, Conceptualization. **Aparicio Chanca-Flores:** Writing – review & editing, Writing – original draft, Visualization, Methodology, Investigation, Formal analysis, Data curation, Conceptualization. **Eugenia Fabián-Arias:** Writing – review & editing, Writing – original draft, Visualization, Investigation, Formal analysis, Data curation, Conceptualization. **Mauro Rafaele-de-la-Cruz:** Writing – review & editing, Writing – original draft, Visualization, Methodology, Investigation, Formal analysis, Data curation, Conceptualization. **Shyla Del-Aguila-Arcentales:** Writing – review & editing, Writing – original draft, Validation, Supervision, Project administration, Methodology, Investigation. **Neal M. Davies:** Writing – review & editing, Writing – original draft, Visualization. **Maria Luz Ortiz de Agui:** Writing – original draft, Visualization, Investigation, Formal analysis, Conceptualization. **Jaime A. Yáñez:** Writing – review & editing, Writing – original draft, Visualization.

## Declaration of competing interest

The authors declare that they have no known competing financial interests or personal relationships that could have appeared to influence the work reported in this paper.
