# Supplementary material for: Evolving attitudes toward online education in Peruvian university students: A quantitative approach
Source: Heliyon. 2024 Apr 30;10(9):e30566. doi: 10.1016/j.heliyon.2024.e30566 (PMC11079248; doi:10.1016/j.heliyon.2024.e30566)
Supplement: Multimedia component 1 [file mmc1.docx]

**Evolving attitudes toward online education in Peruvian university students: a quantitative approach**

**Questionnaire (Spanish version)**

**INFORMACIÓN GENERAL**

Facultad:

Antropología ( ) Ciencias de la Comunicación ( ) Sociología ( ) Trabajo Social ( )

Código de estudiante: ………………..

Ciclo o semestre de estudios: ……………….

Edad: ……

Sexo: Masculino ( ) Femenino ( )

Provincia donde se encuentra: …………………………

Estado civil: Soltero(a) ( ) Casado(a) o conviviente ( ) Divorciado(a) o separado(a) ( ) Viudo(a) ( )

Número de integrantes de la familia con la que convive: ……………

**INSTRUCCIONES:**

Lea atentamente las preguntas y responda con la mayor sinceridad posible, marcando la alternativa que considere conveniente, según el grado de cada una de las preguntas.

**Marque la casilla que usted considere conveniente, teniendo en cuenta las siguientes opciones de respuesta:**

***1. Totalmente en desacuerdo***

***2. En desacuerdo***

***3. Indeciso***

***4. De acuerdo***

***5. Totalmente de acuerdo***

| **Preguntas** | **1** | **2** | **3** | **4** | **5** |
| --- | --- | --- | --- | --- | --- |
| 1. La educación online ayuda a resolver problemas educativos. |  |  |  |  |  |
| 2. La educación online ahorra tiempo y esfuerzo tanto a docentes como a estudiantes. |  |  |  |  |  |
| 3. El acceso a los estudios universitarios está aumentado a través de la educación online. |  |  |  |  |  |
| 4. La educación online me permitirá lograr mejores resultados. |  |  |  |  |  |
| 5. La educación online puede comprometer a los estudiantes más que otras formas de aprendizaje. |  |  |  |  |  |
| 6. La educación online aumenta la calidad de enseñanza - aprendizaje porque integra todos los medios educativos (texto, audio, video y animación). |  |  |  |  |  |
| 7. La educación online aumenta la flexibilidad de la enseñanza - aprendizaje. |  |  |  |  |  |
| 8. La interacción entre estudiantes y docentes mejora a través de la educación online. |  |  |  |  |  |
| 9. El valor pedagógico de una asignatura se puede potenciar a través de tecnologías de la educación online. |  |  |  |  |  |
| 10. La educación online ha creado más problemas que los que ha resuelto. |  |  |  |  |  |
| 11. La educación online no ha tenido impacto positivo en mí. |  |  |  |  |  |
| 12. La educación online nunca reemplazará otras formas de enseñanza y aprendizaje. |  |  |  |  |  |
| 13. Las universidades deberían adoptar cada vez más la educación online para sus estudiantes. |  |  |  |  |  |
| 14. La educación online mejorará mi desempeño académico. |  |  |  |  |  |
| 15. La educación online potenciará mi efectividad para remitir trabajos académicos a tiempo. |  |  |  |  |  |
| 16. La educación online potenciará mi efectividad para reforzar mis conocimientos. |  |  |  |  |  |
| 17. La educación online consume demasiado tiempo al usarla. |  |  |  |  |  |
| 18. La educación online potenciará mi efectividad al realizar investigación. |  |  |  |  |  |
| 19. La educación online me incomoda porque no la entiendo. |  |  |  |  |  |
| 20. La educación online es un proceso deshumanizante del aprendizaje. |  |  |  |  |  |
| 21. La educación online no es efectiva para el aprendizaje de los estudiantes. |  |  |  |  |  |
| 22. Me siento intimidado(a) por la educación online. |  |  |  |  |  |
| 23. Siento angustia cuando debo usar la educación online para mis asignaturas. |  |  |  |  |  |
| 24. Me disgusta la idea de seguir usando la educación online. |  |  |  |  |  |
| 25. Me siento sumamente motivado(a) cuando tomo asignaturas de educación online |  |  |  |  |  |
| 26. Creo que es una buena idea usar la educación online para mis asignaturas. |  |  |  |  |  |
| 27. Encuentro la educación online fácil de usar. |  |  |  |  |  |
| 28. Estoy en contra de la implementación de la educación online, porque impide la interacción cara a cara entre estudiantes y educadores. |  |  |  |  |  |
| 29. Estoy en contra de la educación online, porque conduce al aislamiento de la persona. |  |  |  |  |  |
| 30. Pienso positivamente sobre la educación online. |  |  |  |  |  |
| 31. Planeo participar en futuros cursos de educación online. |  |  |  |  |  |
| 32. El uso de la educación online hace que el aprendizaje sea agradable. |  |  |  |  |  |
| 33. No puedo aprender las asignaturas solo través de la educación online. |  |  |  |  |  |
| 34. Es difícil aprender a través de la educación online. |  |  |  |  |  |
| 35. Es difícil expresar mis pensamientos e ideas mientras envío respuestas en línea. |  |  |  |  |  |
| 36. Es difícil asumir la responsabilidad de mi propio aprendizaje por utilizar educación online. |  |  |  |  |  |
| 37. Es difícil comunicarme efectivamente con mis compañeros(as) usando la educación online. |  |  |  |  |  |
| 38. Los sistemas y plataformas de educación online son fáciles de dominar. |  |  |  |  |  |
| 39. Mi interacción con los contenidos de la educación online no es adecuada. |  |  |  |  |  |
| 40. Aprendo mejor a través del contacto cara a cara con docentes y estudiantes que usando la computadora. |  |  |  |  |  |
| 41. Creo que es mejor leer de una fuente impresa, como un libro o folleto, en lugar de hacerlo desde la pantalla de una computadora. |  |  |  |  |  |
| 42. Encuentro que la educación online es más fácil que usar libros, revistas científicas o tesis en la biblioteca. |  |  |  |  |  |
| 43. Siento que los estudiantes se están volviendo esclavos de la tecnología. |  |  |  |  |  |
| 44. Mi Universidad tiene la tecnología necesaria para brindar educación online. |  |  |  |  |  |
| 45. Mi Universidad tiene un sitio web actualizado para educación online. |  |  |  |  |  |
| 46. Mi Universidad tiene recursos online (revistas y/o libros digitales) para estimular mis actividades de aprendizaje. |  |  |  |  |  |
| 47. Mi Universidad tiene recursos online (revistas y/o libros digitales) para estimular mis actividades de investigación. |  |  |  |  |  |
| 48. Mi Universidad tiene asistencia técnica cuando busco ayuda del servicio de soporte. |  |  |  |  |  |
| 49. Mi Universidad no tiene el financiamiento para adquirir hardware y software actualizados y necesarios. |  |  |  |  |  |
| 50. Mi Facultad tiene docentes capacitados para la enseñanza - aprendizaje online. |  |  |  |  |  |
| 51. Los estudiantes de mi Facultad están motivados por la adopción de la educación online. |  |  |  |  |  |
| 52. Los estudiantes de mi Facultad prefieren las maneras tradicionales de enseñanza e investigación. |  |  |  |  |  |
| 53. Me siento inseguro de mi capacidad de utilizar las herramientas de la educación online. |  |  |  |  |  |
| 54. Me estreso por las conexiones lentas a Internet mientras recibo educación online. |  |  |  |  |  |
| 55. Me siento presionado(a) por mis docentes para usar la educación online en mis actividades de investigación. |  |  |  |  |  |
| 56. Me siento presionado(a) por mis docentes para usar la educación online en mis actividades de aprendizaje. |  |  |  |  |  |
| 57. Me siento presionado(a) por mis compañeros(as) para usar la educación online. |  |  |  |  |  |
| 58. Me siento estresado(a) porque mis equipos tecnológicos son poco confiables para usar la educación online. |  |  |  |  |  |
| 59. La educación online debe seguir ofreciéndose para llegar a los estudiantes que viven en lugares lejanos. |  |  |  |  |  |
| 60. La educación online debe seguir ofreciéndose para reducir el estrés relacionado al viaje de los docentes y estudiantes. |  |  |  |  |  |
| 61. La educación online debe seguir ofreciéndose para permitir que los estudiantes casados equilibren las exigencias familiares y del estudio. |  |  |  |  |  |
| 62. La educación online debe seguir ofreciéndose para permitir a los estudiantes que trabajan estudiar desde casa. |  |  |  |  |  |
| 63. La educación online es la mejor alternativa para afrontar la actual situación de crisis social. |  |  |  |  |  |

**Muchas gracias.**

**Evolving attitudes toward online education in Peruvian university students: a quantitative approach**

**Questionnaire (English version)**

**GENERAL INFORMATION**

Faculty:

Anthropology ( ) Communication Sciences ( ) Sociology ( ) Sociology ( ) Social Work ( )

Student code: ....................

Cycle or semester of studies: ...................

Age: ......

Sex: Male ( ) Female ( )

Province where you are: ..............................

Marital status: Single ( ) Married or cohabitating ( ) Divorced or separated ( ) Widowed ( )

Number of family members with whom you live: ...............

**INSTRUCTIONS**

Read the questions carefully and answer as honestly as possible, ticking the alternative you consider appropriate, according to the grade of each question.

**Please tick the box that you consider appropriate, considering the following answer options:**

***1. Strongly disagree***

***2.*** ***Disagree***

***3.*** ***Undecided***

***4.*** ***Agree***

***5.*** ***Strongly agree***

| **Questions** | **1** | **2** | **3** | **4** | **5** |
| --- | --- | --- | --- | --- | --- |
| 1. Online education helps solve educational problems. |  |  |  |  |  |
| 2. Online education saves time and effort for both teachers and students. |  |  |  |  |  |
| 3. Access to university studies is increased through online education. |  |  |  |  |  |
| 4. Online education will allow me to achieve better results. |  |  |  |  |  |
| 5. Online education can engage students more than other forms of learning. |  |  |  |  |  |
| 6. Online education increases the quality of teaching - learning because it integrates all educational media (text, audio, video and animation). |  |  |  |  |  |
| 7. Online education increases the flexibility of teaching - learning. |  |  |  |  |  |
| 8. The interaction between students and teachers improves through online education. |  |  |  |  |  |
| 9. The pedagogical value of a subject can be enhanced through online education technologies. |  |  |  |  |  |
| 10. Online education has created more problems than it has solved. |  |  |  |  |  |
| 11. Online education has not had a positive impact on me. |  |  |  |  |  |
| 12. Online education will never replace other forms of teaching and learning. |  |  |  |  |  |
| 13. Universities should increasingly adopt online education for their students. |  |  |  |  |  |
| 14. Online education will improve my academic performance. |  |  |  |  |  |
| 15. Online education will enhance my effectiveness in submitting academic work on time. |  |  |  |  |  |
| 16. Online education will enhance my effectiveness to reinforce my knowledge. |  |  |  |  |  |
| 17. Online education consumes too much time when using it. |  |  |  |  |  |
| 18. Online education will enhance my effectiveness when conducting research. |  |  |  |  |  |
| 19. Online education makes me uncomfortable because I don't understand it. |  |  |  |  |  |
| 20. Online education is a dehumanizing learning process. |  |  |  |  |  |
| 21. Online education is not effective for student learning. |  |  |  |  |  |
| 22. I feel intimidated by online education. |  |  |  |  |  |
| 23. I feel anguish when I have to use online education for my subjects. |  |  |  |  |  |
| 24. I dislike the idea of continuing to use online education. |  |  |  |  |  |
| 25. I feel extremely motivated when I take online education subjects |  |  |  |  |  |
| 26. I think it is a good idea to use online education for my subjects. |  |  |  |  |  |
| 27. I find online education easy to use. |  |  |  |  |  |
| 28. I am against the implementation of online education, because it prevents face-to-face interaction between students and educators. |  |  |  |  |  |
| 29. I am against online education, because it leads to the isolation of the person. |  |  |  |  |  |
| 30. I think positively about online education. |  |  |  |  |  |
| 31. I plan to participate in future online education courses. |  |  |  |  |  |
| 32. The use of online education makes learning enjoyable. |  |  |  |  |  |
| 33. I cannot learn the subjects only through online education. |  |  |  |  |  |
| 34. It is difficult to learn through online education. |  |  |  |  |  |
| 35. It is difficult to express my thoughts and ideas while submitting responses online. |  |  |  |  |  |
| 36. It is difficult to take responsibility for my own learning by using online education. |  |  |  |  |  |
| 37. It is difficult to communicate effectively with my classmates using online education. |  |  |  |  |  |
| 38. Online education systems and platforms are easy to master. |  |  |  |  |  |
| 39. My interaction with the content of online education is not adequate. |  |  |  |  |  |
| 40. I learn better through face-to-face contact with teachers and students than using the computer. |  |  |  |  |  |
| 41. I think it is better to read from a printed source, such as a book or pamphlet, rather than from a computer screen. |  |  |  |  |  |
| 42. I find online education easier than using books, scientific journals or theses in the library. |  |  |  |  |  |
| 43. I feel that students are becoming slaves to technology. |  |  |  |  |  |
| 44. My University has the necessary technology to provide online education. |  |  |  |  |  |
| 45. My University has an updated website for online education. |  |  |  |  |  |
| 46. My University has online resources (magazines and/or digital books) to stimulate my learning activities. |  |  |  |  |  |
| 47. My University has online resources (magazines and/or digital books) to stimulate my research activities. |  |  |  |  |  |
| 48. My University has technical assistance when I seek help from the support service. |  |  |  |  |  |
| 49. My University does not have the financing to acquire updated and necessary hardware and software. |  |  |  |  |  |
| 50. My Faculty has teachers trained in online teaching and learning. |  |  |  |  |  |
| 51. The students at my Faculty are motivated by the adoption of online education. |  |  |  |  |  |
| 52. Students at my Faculty prefer traditional ways of teaching and research. |  |  |  |  |  |
| 53. I feel insecure about my ability to use the tools of online education. |  |  |  |  |  |
| 54. I get stressed about slow internet connections while receiving online education. |  |  |  |  |  |
| 55. I feel pressured by my teachers to use online education in my research activities. |  |  |  |  |  |
| 56. I feel pressured by my teachers to use online education in my learning activities. |  |  |  |  |  |
| 57. I feel pressured by my peers to use online education. |  |  |  |  |  |
| 58. I feel stressed because my technological equipment is unreliable to use online education. |  |  |  |  |  |
| 59. Online education must continue to be offered to reach students living in distant places. |  |  |  |  |  |
| 60. Online education should continue to be offered to reduce travel-related stress for teachers and students. |  |  |  |  |  |
| 61. Online education should continue to be offered to allow married students to balance family and study demands. |  |  |  |  |  |
| 62. Online education should continue to be offered to allow working students to study from home. |  |  |  |  |  |
| 63. Online education is the best alternative to face the current situation of social crisis. |  |  |  |  |  |

Thank you.
